# Selinexor (KPT-330) demonstrates anti-tumor efficacy in preclinical models of triple-negative breast cancer

**DOI:** 10.1186/s13058-017-0878-6

**Published:** 2017-08-15

**Authors:** Natalia Paez Arango, Erkan Yuca, Ming Zhao, Kurt W. Evans, Stephen Scott, Charissa Kim, Ana Maria Gonzalez-Angulo, Filip Janku, Naoto T. Ueno, Debu Tripathy, Argun Akcakanat, Aung Naing, Funda Meric-Bernstam

**Affiliations:** 1 Department of Surgical Oncology, The University of Texas MD Anderson Cancer Center, 1400 Pressler Street, Houston, TX 77030 USA; 2 Department of Investigational Cancer Therapeutics, The University of Texas MD Anderson Cancer Center, 1400 Holcombe Boulevard, Unit 455, Houston, TX 77030 USA; 3 Department of Genetics, The University of Texas MD Anderson Cancer Center, 1515 Holcombe Blvd, Houston, TX USA; 4 Department of Breast Medical Oncology, The University of Texas MD Anderson Cancer Center, 1515 Holcombe Blvd, Houston, TX USA; 5 The Sheikh Bin Zayed Al Nahyan Institute for Personalized Cancer Therapy, The University of Texas MD Anderson Cancer Center, 1400 Pressler Boulevard, Unit 455, Houston, TX 77030 USA; 6 Department of Breast Surgical Oncology, The University of Texas MD Anderson Cancer Center, 1400 Pressler Boulevard, Unit 455, Houston, TX 77030 USA

**Keywords:** Breast cancer, Selinexor, XPO1, PDX, TNBC

## Abstract

**Background:**

Selinexor (KPT-330) is an oral agent that has been shown to inhibit the nuclear exporter XPO1. Given the pressing need for novel therapies for triple-negative breast cancer (TNBC), we sought to determine the antitumor effects of selinexor in vitro and in vivo.

**Methods:**

Twenty-six breast cancer cell lines of different breast cancer subtypes were treated with selinexor in vitro. Cell proliferation assays were used to measure the half-maximal inhibitory concentration (IC_50_) and to test the effects in combination with chemotherapy. In vivo efficacy was tested both as a single agent and in combination therapy in TNBC patient-derived xenografts (PDXs).

**Results:**

Selinexor demonstrated growth inhibition in all 14 TNBC cell lines tested; TNBC cell lines were more sensitive to selinexor (median IC_50_ 44 nM, range 11 to 550 nM) than were estrogen receptor (ER)-positive breast cancer cell lines (median IC_50_ > 1000 nM, range 40 to >1000 nM; *P* = 0.017). In multiple TNBC cell lines, selinexor was synergistic with paclitaxel, carboplatin, eribulin, and doxorubicin in vitro*.* Selinexor as a single agent reduced tumor growth in vivo in four of five different TNBC PDX models, with a median tumor growth inhibition ratio (T/C: treatment/control) of 42% (range 31 to 73%) and demonstrated greater antitumor efficacy in combination with paclitaxel or eribulin (average T/C ratios of 27% and 12%, respectively).

**Conclusions:**

Collectively, these findings strongly suggest that selinexor is a promising therapeutic agent for TNBC as a single agent and in combination with standard chemotherapy.

**Electronic supplementary material:**

The online version of this article (doi:10.1186/s13058-017-0878-6) contains supplementary material, which is available to authorized users.

## Background

Normal cell homeostasis depends on the cell’s ability to compartmentalize proteins. During carcinogenesis, nuclear export is disrupted and as consequence multiple tumor-suppressor proteins that usually trigger apoptosis and cell cycle arrest in the nucleus are transported to the cytoplasm where they are subsequently targeted for degradation [1]. The nuclear exporter Exportin1 or CRM1 (XPO1) is one of at least seven exportins that mediate the transport of over 200 proteins, including several key regulators of the cell cycle (e.g., p53, STAT3, survivin, FoxO3a, BRCA1, and others) [2, 3]. XPO1 interacts with cargo proteins through a nuclear export sequence (NES), and it is dependent on the small GTPase protein Ran in its guanine triphosphate (GTP)-bound form, which binds to XPO1 together with export cargos [4].

Increased XPO1 expression has been linked to poor prognosis in multiple solid and hematologic malignancies [5–7], and therefore inhibition of XPO1 is being pursued as a promising target for cancer therapy. Selinexor (KPT-330) is an oral agent that has been shown to inhibit XPO1 [8] and is currently in clinical trials for treatment of hematologic and solid malignancies, with promising results.

Approximately 15% of all breast cancers are categorized as triple-negative breast cancer (TNBC) [9]. Given that there is no specific targeted therapy, systemic chemotherapy is usually the first line of treatment [10]. However, currently available chemotherapy regimens for advanced TNBC often fail to achieve disease control. Given the poor prognosis for patients with TNBC, there is a pressing need for novel therapies. XPO1 mediates nuclear export of several proteins that have been linked to TNBC [11], and there have been efforts to show breast cancer growth inhibition by several selective inhibitors of nuclear export [12]. We therefore sought to determine the antitumor effects of selinexor on breast cancer by testing multiple cell lines in vitro and in multiple patient-derived xenograft (PDX) models in vivo, with a particular focus on TNBC. From there, we sought to determine the best possible combination with cytotoxic chemotherapies.

## Methods

### Cell lines and culture conditions

Twenty-six breast cancer cell lines were obtained from the American Tissue Culture Collection (ATCC, Manassas, VA, USA): BT-20, BT-474, BT-483, BT-549, CAMA-1, HCC-38, HCC-1143, HCC-1395, HCC-1419, HCC-1569, HCC-1806, HCC-1937, HCC-1954, HCC-70, MCF-7, MDA-MB-134vi, MDA-MB-157, MDA-MB-175, MDA-MB-231, MDA-MB-361, MDA-MB-436, MDA-MB-453, MDA-MB-468, SK-BR-3, SUM-159PT, and T-47D. Cells were cultured in Dulbecco’s modified Eagle’s medium/F-12 supplemented with 10% fetal bovine serum at 37 °C and humidified 5% CO_2_.

### Drugs and reagents

Selinexor was obtained from Karyopharm Therapeutics Inc. as a generous gift. For in vivo experiments, selinexor was prepared in 0.6% w/v Pluronic F-68 surfactant and 0.6% w/v PVP K-29/32 polymer. Paclitaxel, carboplatin, and gemcitabine were purchased from Selleck Chemicals. Doxorubicin and eribulin were acquired from the MD Anderson Cancer Center pharmacy. Dimethyl sulfoxide (DMSO) was from Sigma-Aldrich. For in vitro studies, all drugs were dissolved in DMSO.

### Cell proliferation assays

Cells were seeded in 96-well plates at densities of 3000 to 6000 cells per well depending on the growth characteristics of each cell line. After cells had adhered overnight, titrating concentrations of the designated drug were added to the wells in triplicate and incubated at 37 °C for 72 hours. Cell viability was then measured using sulforhodamine B (SRB) staining. The half-maximal inhibitory concentration (IC_50_) was determined based on a dose-response curve generated using GraphPad Prism software, v6.05. All experiments were performed at least three times.

### Colony formation assay

Cells were plated at a density of 2 × 10^3^ cells on 60-mm plates in triplicate for each treatment group. Cells were treated the next day with the indicated concentrations of selinexor, paclitaxel, eribulin, vehicle control (DMSO), or a combination of selinexor and paclitaxel or eribulin for 2 weeks. The culture medium was changed every 7 days. The colonies were then fixed in 10% formalin and stained with 0.05% crystal violet in 25% methanol. Percent surface area was measured using NIH ImageJ software, v.1.48.

### Western blot analysis

Cells were washed with cold PBS and lysed in Laemmli buffer. The protein was quantified using the Pierce BCA protein assay kit (ThermoFisher) before loading onto the gel. After SDS-PAGE, the protein was transferred to a 0.2-μm nitrocellulose membrane (Bio-Rad Laboratories). Membranes were blocked with 0.1% casein in Tris-buffered saline. Immunoblotting was performed with anti-β-catenin, anti-cyclin D1, anti-XIAP, and anti-survivin (Cell Signaling Technology), anti-β-actin, XPO1 (Santa Cruz Biotechnology), followed by secondary antibody anti-rabbit IgG Alexa fluor 680 (Sigma). The immunoblots were visualized using the Odyssey IR imaging system (Li-Cor Biosciences). Image Studio software, v4.0 was used to analyze the bands. Each band was normalized to its respective β-actin band. Representative blots of at least two independent experiments are shown. Results were analyzed using Student’s unpaired *t* test, with two-tailed *P* values. Values are presented as mean ± SEM.

### Apoptosis and cell cycle assays

Cells were plated and treated the following day with DMSO or with selinexor and/or paclitaxel in triplicate. After 72 hours, both floating cells and attached cells were collected. Apoptosis was identified by using the annexin V apoptosis kit (Roche cat. no. 11858777001) according to the manufacturer’s protocol. The samples were analyzed by flow cytometry at the Flow Cytometry and Cellular Imaging Core Facility at MD Anderson. For cell cycle analysis, cells were collected after 72 hours of treatment, and DNA content was determined with flow cytometry using propidium iodide (Roche cat. no. 11348639001) following the manufacturer’s protocol. Each experiment was performed at least three times.

### In vivo studies

All animal experiments were approved by the Institutional Animal Care and Use Committee of MD Anderson. TNBC PDX models have been previously described [13]. Tumors were implanted into female BALB/c *nu*/*nu* mice, 6 to 8 weeks old, under isoflurane anesthesia. A skin incision (approximately 0.3 cm) was made with a subcutaneous pocket on the mid back. One tumor piece (approximately 27 mm^3^) was inserted into a pocket and the skin was then closed. The mice were treated when the tumor diameter reached at least 200 mm^3^. The mice were killed when the diameter reached 1.5 cm, and the individual relative tumor volume (RTV) was measured. RTV was defined as Vx/V1, where Vx is the volume in mm^3^ at a given time and V1 is the volume at the start of treatment [14, 15]. The statistical analyses were performed by comparing RTV in the treatment arms with RTV in the vehicle arm. Tumor growth inhibition ratios (T/C: treatment/control) were calculated using the formula:

[(Median tumor volume of treated group)/(Median tumor volume of control group)] × 100.

Activity was defined as a T/C ratio <40% [16, 17].

The BCX 10 mice were treated with vehicle or selinexor via oral gavage 12.5 mg/kg twice a week (2 weeks on, 1 week off); the BCX 6 and BCX 11 mice were treated with selinexor via oral gavage 12.5 mg/kg once a week. The dose for these experiments was decreased to model a clinical trial that was being run in parallel. Mice in the three models were also treated with paclitaxel 10 mg/kg intravenously weekly (2 weeks on, 1 week off), eribulin 1 mg/kg intravenously weekly (2 weeks on, 1 week off), carboplatin 75 mg/kg intraperitoneally weekly (1 week on, 2 weeks off), or a combination of selinexor with each individual drug.

## Results

### Selinexor has potent antitumor efficacy in breast cancer cells in vitro

**Fig. 1:**
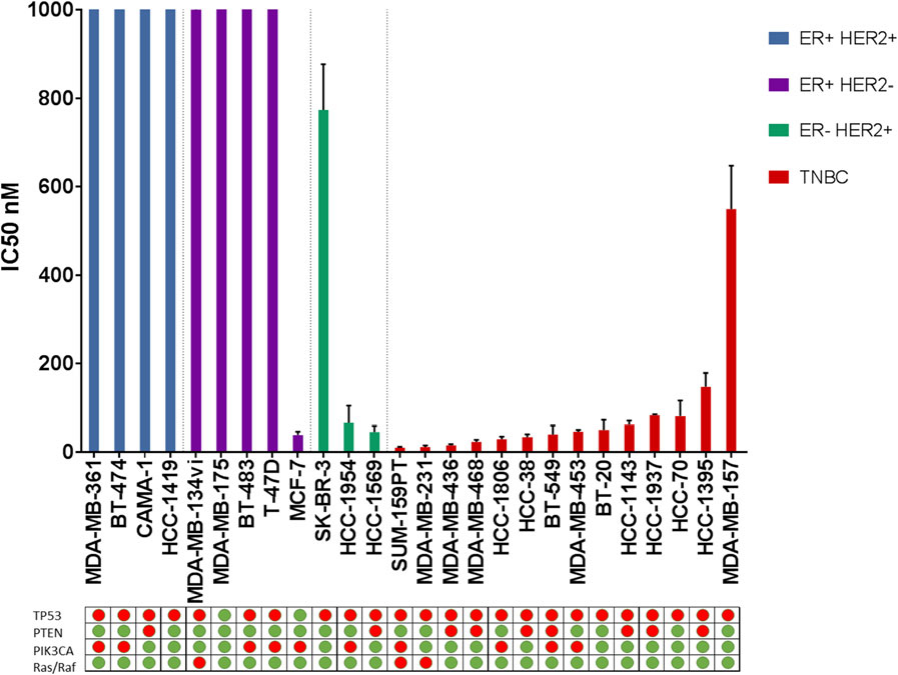
Effects of selinexor on cell proliferation in vitro*.* Twenty-six breast cancer cell lines representing varying hormone receptor status were treated with selinexor at 10 concentrations based on a fivefold dilution series (range 0 to 100 μM). Cell growth was measured after 72 hours of treatment using the sulforhodamine B assay, and half-maximal inhibitory concentration (*IC*_*50*_) was then calculated using isobologram curves, with an overall median IC_50_ of 66 nM (range 11 to >1000 nM). Sensitivity was defined as IC_50_ < 1000 nM, a physiologically achievable dose based on previous phase I studies. TP53, PTEN, PIK3CA, and Ras/Raf status is shown. *Green circle* wild type, *red circle* mutation/deletion or frameshift. *ER* estrogen receptor, *HER2* human epidermal growth factor receptor, *TNBC* triple-negative breast cancer

To test the effects of selinexor in breast cancer, we selected 26 breast cancer cell lines representing different subtypes that included TNBC and estrogen receptor (ER)-positive (ER+) and human epidermal growth factor receptor (HER2) + cell lines, with various genomic alterations including *PIK3CA*, *TP53*, and *PTEN* mutations as described in Additional file 1. Cell growth was measured after 72 hours of treatment using the SRB colorimetric assay. Sensitivity was evaluated by calculating the IC_50_ using isobologram curves. The sensitivities among the cell lines varied, with an overall median IC_50_ of 66 nM (range 11 to >1000 nM) (Fig. 1). Sensitivity was defined as <1000 nM, which is a physiologically achievable dose based on previous phase I studies [18]. All 14 of the TNBC cell lines tested were significantly more sensitive to selinexor (median IC_50_ of 44 nM, range 11 to 550 nM) than were ER+ cell lines (median IC_50_ of >1000 nM, range 40 to >1000 nM) (*P* = 0.017).

**Fig. 2:**
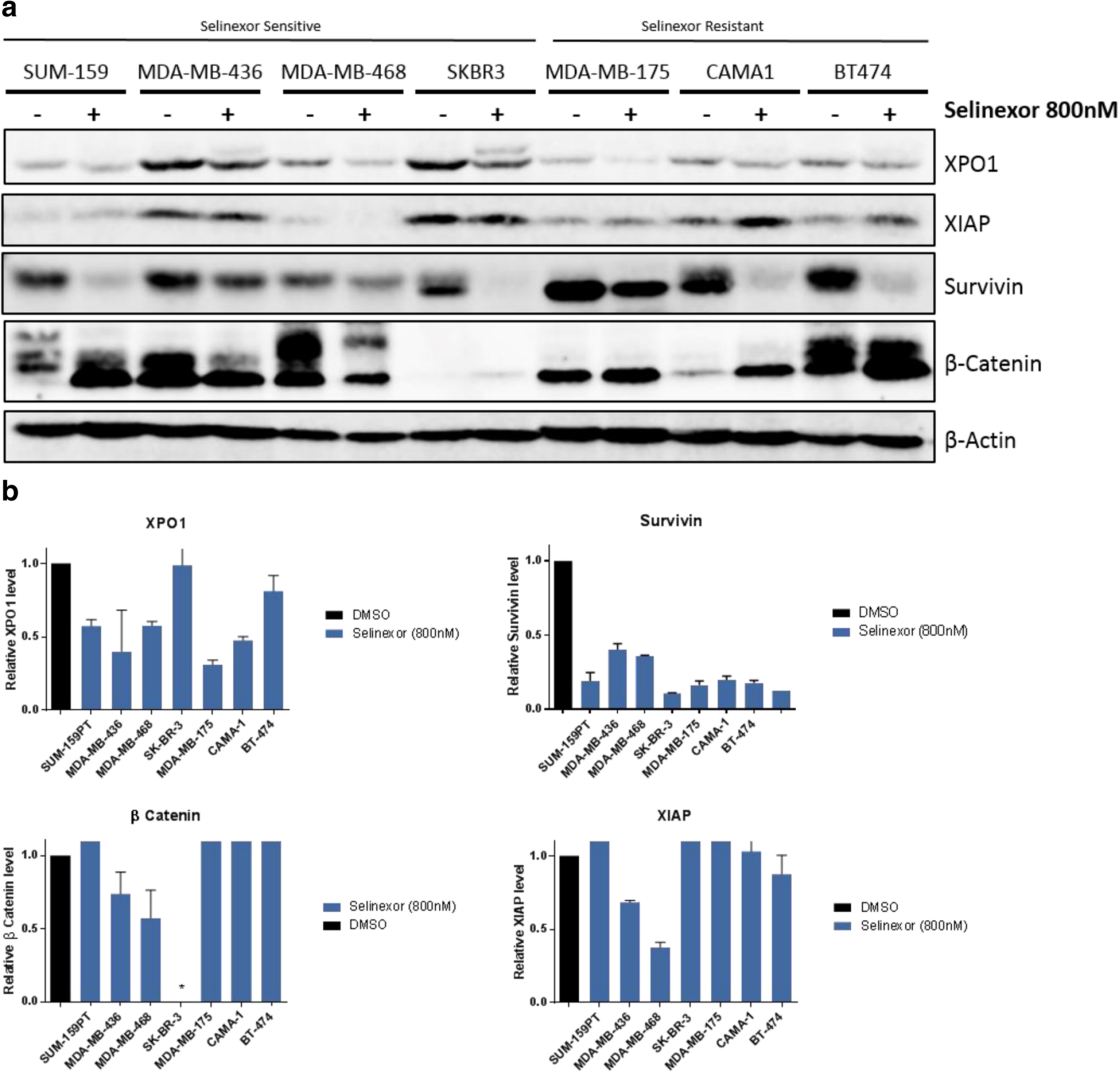
Effects of selinexor in breast cancer models. **a** Seven breast cancer cell lines were treated with vehicle or 800 nM selinexor, a physiologically achievable dose. Cells were lysed and blotted with the indicated antibodies. **b** The bands were quantified and normalized to their respective β-actin controls. Values are shown as relative protein levels vs vehicle control of cell lines treated with selinexor

To assess the effects of selinexor on XPO1 expression, we chose an array of breast cancer cell lines representing varying hormone receptor status and representing different levels of sensitivity to selinexor. Seventy-two hours after treatment with or without 800 nM selinexor (a physiologically achievable concentration [18]), cells were lysed and immunoblotted. The intensities of XPO1 bands were quantified and normalized to their respective β-actin bands. Selinexor reduced the levels of XPO1 and of survivin in all cell lines. XIAP (an inhibitor of apoptosis protein) and β-catenin (a known regulator of survivin [19, 20]) were lower in MDA-MB-436 and MDA-MB-468, both TNBC cell lines that are sensitive to selinexor (Fig. 2). These effects were more robust when the dose of selinexor was increased to 5 μM (Additional file 2).

### Selinexor in combination with standard chemotherapy enhanced antitumor efficacy in vitro

**Fig. 3:**
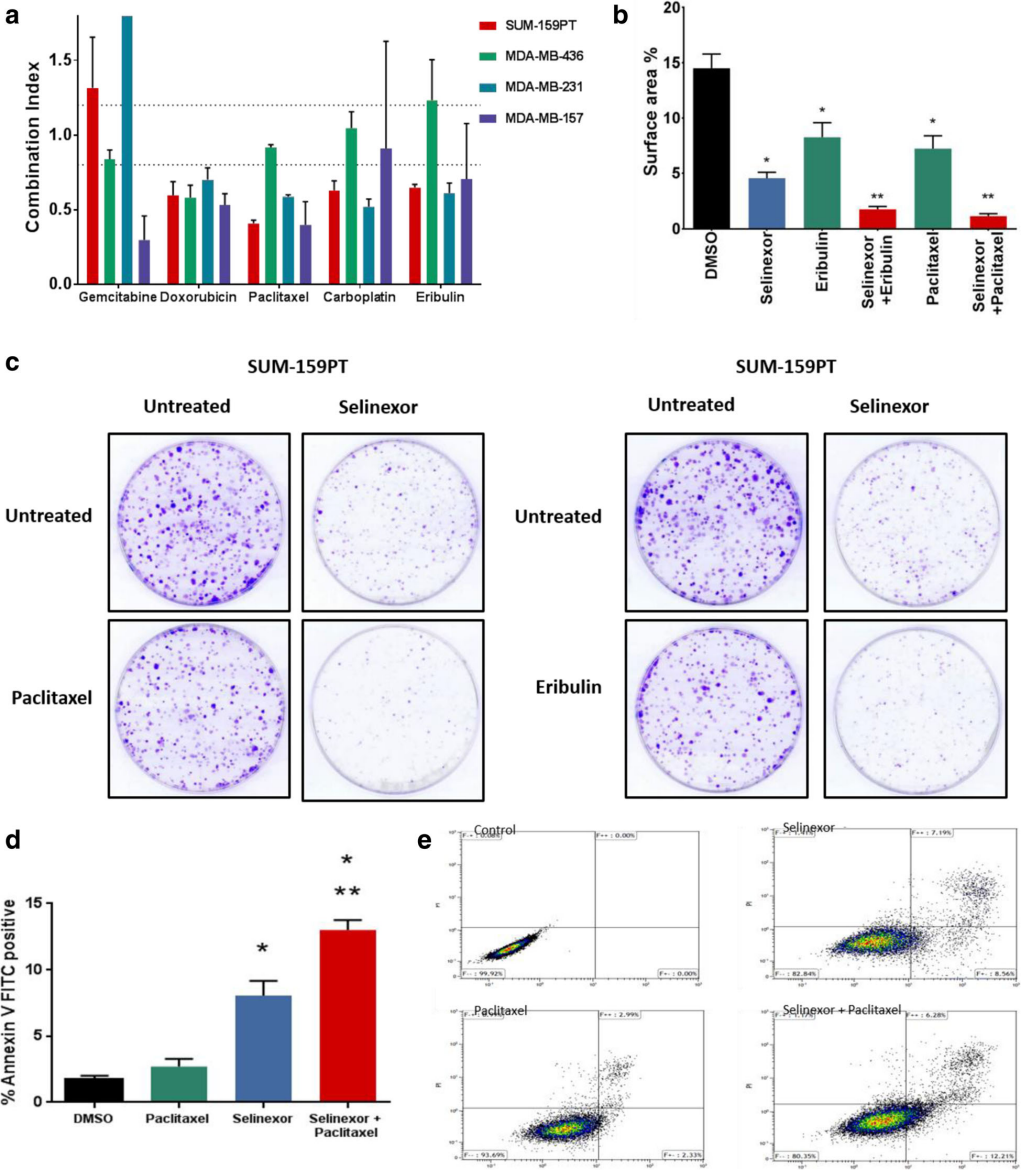
Effects of selinexor in combination with standard chemotherapy in vitro*.* **a** Four different triple-negative breast cancer cell lines were treated with selinexor in combination with paclitaxel, doxorubicin, gemcitabine, carboplatin, and eribulin. Cell growth was measured after 72 hours of treatment using the Sulforhodamine B (SRB) assay, and the combination index (CI) was then calculated using the method of Chou and Talalay [20]. A CI value <0.8 indicates synergism, a value equal to 1 indicates addition, and a CI significantly >1.2 indicates antagonism. **b**, **c** SUM-159PT cells were trypsinized, counted, and plated at a density of 2 × 10^3^ cells in 60-mm plates in triplicate for each treatment group. Cells were treated for 2 weeks with vehicle, selinexor (50 nM), paclitaxel (0.5 nM), or eribulin (1 nM) or in combinations of selinexor with paclitaxel and selinexor with eribulin. Colonies were then fixed and stained with crystal violet. Percent surface area was calculated using NIH Image J v.1.48 software. Values are presented as mean ± SEM (*P < 0.005 vs control, **P < 0.001 combination paclitaxel vs selinexor alone, *P* = 0.0013 combination eribulin vs selinexor alone). **d**, **e** SUM-159PT cells were treated with 0.5 nM paclitaxel alone, 400 nM selinexor alone, and the combination of both. After 72 hours, annexin-V-positive cells were determined by fluorescence-activated cell sorting FACS analysis. (*P < 0.001 selinexor alone vs vehicle control, **P = 0.004 combination vs selinexor alone). Each individual experiment was performed in triplicate. *DMSO* dimethyl sulfoxide, *FITC*, fluorescein isothiocyanate

To determine the effects of selinexor in combination with standard chemotherapy, we selected several commonly used agents: paclitaxel (a microtubule stabilizer), carboplatin (DNA-binding alkylating agent), eribulin (microtubule inhibitor), doxorubicin (topoisomerase inhibitor), and gemcitabine (pyrimidine analogue), all with various mechanisms of action. Four TNBC cell lines with different genomic backgrounds were studied: SUM-159PT (*HRAS*, *PIK3CA*, and *TP53* mutant), MDA-MB-436 (*PTEN* and *BRCA* mutant), MDA-MB-231 (*KRAS* and *TP53* mutant), and MDA-MD-157 (*TP53* mutant); all cell lines were XPO1 wild-type. Cells were treated with serial concentration dilutions of selinexor in combination with serial concentration dilutions of the various chemotherapy agents. After 72 hours of treatment, growth inhibition was assessed using the SRB assay and IC_50_ was calculated for single-agent treatment alone and the combinations. Combination index (CI) values were then calculated using the method of Chou and Talalay, where a CI value <0.8 indicates synergism, a CI of 0.8 to 1.2 indicates addition, and a CI >1.2 indicates antagonism [21, 22]. Synergistic effects were found for all four cell lines when selinexor was combined with doxorubicin and paclitaxel and for SUM-159PT and MDA-MB-231 when combined with carboplatin or eribulin (Fig. 3a).

Using the colony formation assay, we then assessed the effects of selinexor in combination with paclitaxel and with eribulin. The TNBC cell line SUM-159PT was treated with either vehicle control (DMSO), 50 nM selinexor, 0.5 nM paclitaxel, 1 nM eribulin, or with a combination of selinexor with paclitaxel or selinexor with eribulin. After 2 weeks of treatment, cell colonies were stained with crystal violet. Plates were then scanned and the percentage of stained surface area was quantified (Fig. 3b and c). Treatment with selinexor in combination with paclitaxel and in combination with eribulin resulted in a dramatic decline in cell growth compared with treatment with either drug alone (*P* < 0.001 for combination paclitaxel vs selinexor alone and *P* = 0.0013 for combination eribulin vs selinexor alone).

Once we established that there was significant synergy between selinexor and paclitaxel, we wanted to determine whether apoptosis was induced. We treated SUM-159PT cells with selinexor, paclitaxel, or selinexor in combination with paclitaxel for 72 hours with a physiologically achievable dose of the compounds. To assess for apoptosis, the harvested cells were analyzed by fluorescence-activated cell sorting (FACS) using the annexin V apoptosis kit. Notably, our results showed an increased number of annexin-V-positive cells when treated with selinexor alone compared with vehicle control (*P* < 0.001). The percentage of cells that were positive for annexin V was significantly higher when selinexor was used in combination with paclitaxel compared with vehicle control (*P* < 0.001) and compared with selinexor alone (*P* = 0.004) (Fig. 3d and e). In a similar fashion, cell cycle analysis was performed on SUM-159PT and MDA-MB-468 cells. The results showed a significantly greater number of cells in sub-G1 phase (*P* = 0.003) when selinexor was used in combination with paclitaxel compared with either treatment alone, as shown in Additional file 3.

### Selinexor has in vivo efficacy in TNBC PDX models

**Fig. 4:**
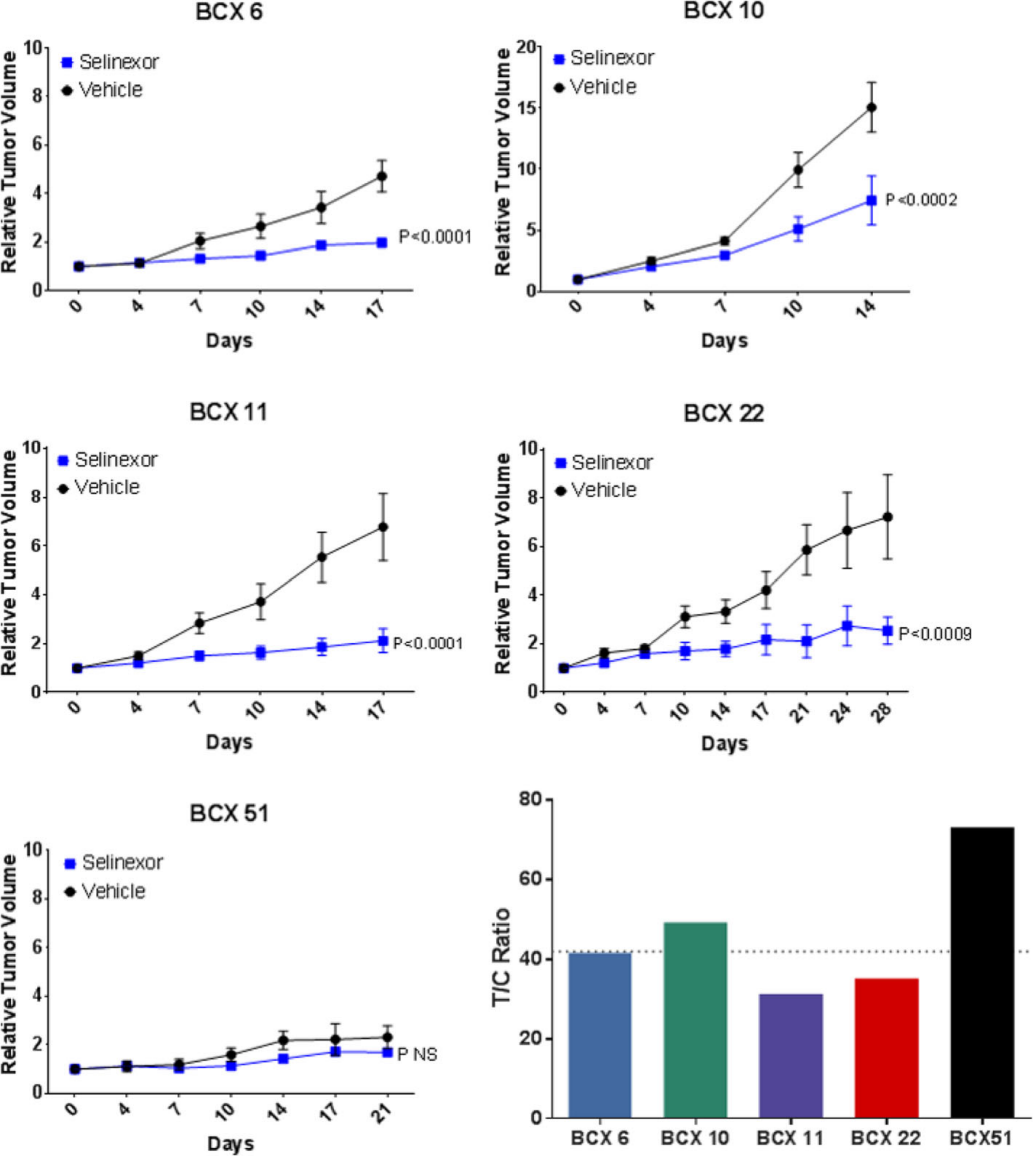
Effects of selinexor on tumor growth of triple-negative breast cancer (TNBC) patient-derived xenograft (PDX) models. Mice bearing BCX 6, BCX 10, BCX 11, BCX 22, and BCX 51 TNBC patient-derived xenografts were treated with vehicle or selinexor 12.5 mg/kg twice a week. Values are presented as mean ± SEM of relative tumor volume. The tumor volumes at the conclusion of experiment were compared with vehicle, and data were analyzed by two-way analysis of variance to determine statistical significance. Tumor growth inhibition (*T/C*) ratios were calculated using the formula: [(Median tumor volume of treated group)/(Median tumor volume of control group)] × 100. Activity was defined as a T/C % ratio <40%

To test the effects of selinexor in vivo, we selected five different TNBC PDX models: BCX 6, BCX 10, BCX 11, BCX 22, and BCX 51, with varying status for *TP53* and *PIK3CA* [13] and gene expression subtypes. Molecular characteristics of these PDX models are listed in Additional file 4 (detailed manuscript on PDXs under review). Mice implanted with these PDX models were treated either with vehicle or selinexor via oral gavage 12.5 mg/kg twice a week (2 weeks on and 1 week off). We observed that, in four of five PDX models, mice treated with selinexor had significantly lower tumor growth (*P* < 0.001), with a median T/C score of 42% (range 31 to 73%) (Fig. 4). The mice tolerated the drug well, with no weight loss during treatment (Additional file 5).

**Fig. 5:**
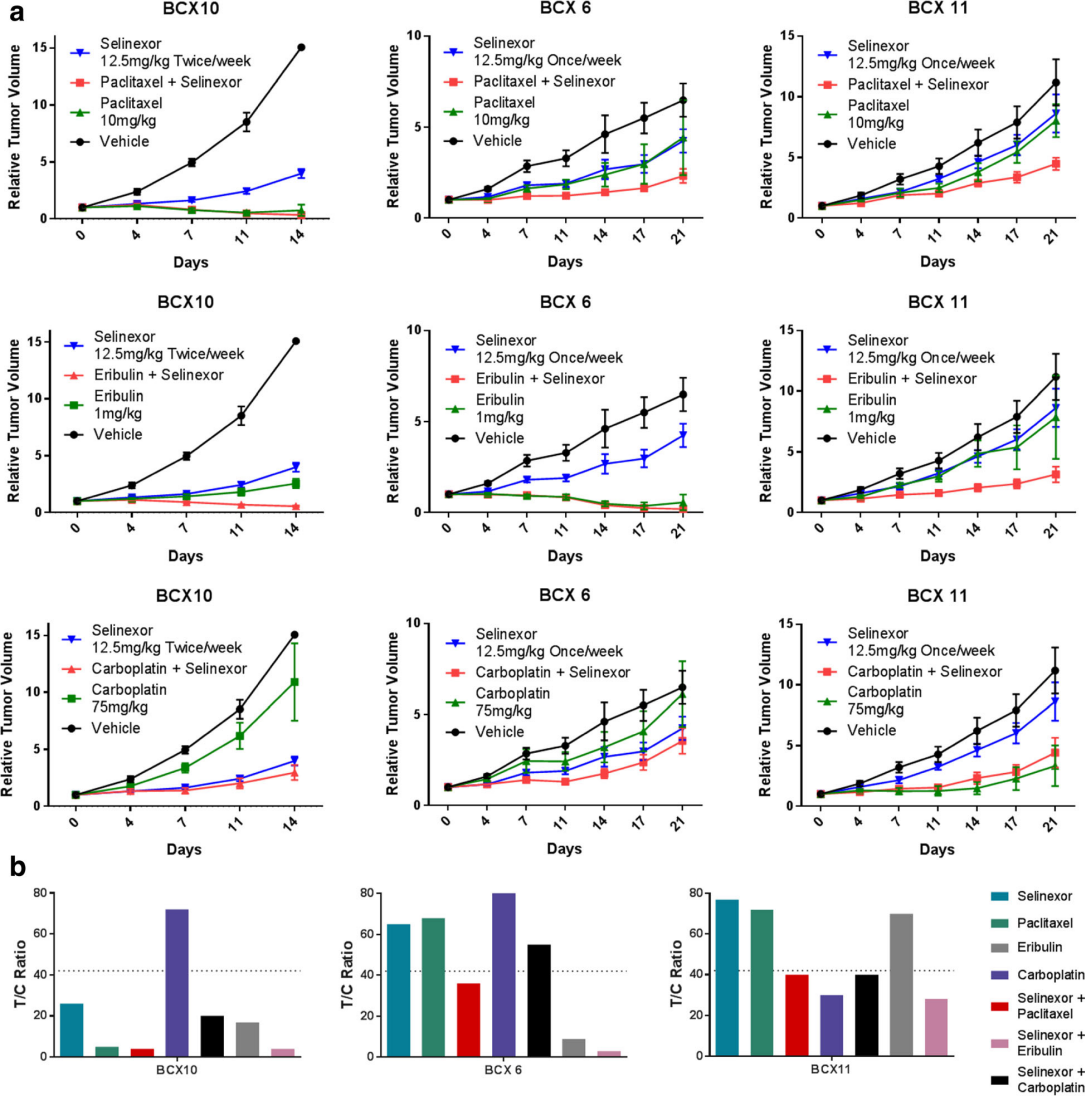
Effects of selinexor on tumor growth in combination with standard chemotherapy. Mice bearing three different triple-negative breast cancer patient-derived xenografts were treated with vehicle, selinexor 12.5 mg/kg twice a week for BCX 10 and once weekly for BCX 6 and BCX 11, paclitaxel 10 mg/kg weekly, eribulin 1 mg/kg, carboplatin 75 mg/kg weekly, and combinations of selinexor with each chemotherapy agent. **a** Values are presented as mean ± SEM. The tumor volumes at the conclusion of the experiment were compared with vehicle, and the data were analyzed by two-way analysis of variance to determine statistical significance. **b** Tumor growth inhibition (*T/C*) ratios were calculated using the formula: [(Median tumor volume of treated group)/(Median tumor volume of control group)] × 100. Activity was defined as a T/C % ratio <40%

Next, we compared the effects of selinexor in vivo in combination with standard chemotherapy agents, including paclitaxel, eribulin, and carboplatin, on BCX 6, BCX 10, and BCX 11 models, all with various degrees of sensitivity to these agents (Fig. 5). In vivo, BCX 10 and BCX 6 were relatively resistant to carboplatin, and although the combination with selinexor was significantly more effective than carboplatin alone (*P* < 0.02), it was not significantly more effective than selinexor alone. However, selinexor in combination with paclitaxel or eribulin had significant antitumor efficacy, with T/C ratios <40% for all three PDX models. For BCX 11, selinexor in combination with paclitaxel compared with either agent alone was significantly more effective (*P* < 0.003), and selinexor in combination with eribulin was significantly more effective than either agent alone in BCX 10 (*P* < 0.001), and BCX 11 (*P* = 0.005).

## Discussion

TNBC occurs in approximately 15 to 20% of patients with breast cancer and is associated with an unfavorable prognosis [10]. Although patients with TNBC who achieve complete pathological response have better survival [23], patients who have significant residual disease are at high risk of relapse and have limited options upon recurrence. Therefore there is a great need for superior therapy options for TNBC. Here we showed the antitumor efficacy of selinexor, as a novel approach for TNBC therapy.

XPO1 compartmentalizes tumor suppressors and cell cycle regulators, which are dependent on location to exert their apoptotic/proliferative functions [24]. Many of these have been linked to the elusive molecular network in TNBC [11], providing a rationale for exploring XPO1 inhibition as a potential target for cancer therapy. Our findings have established the antitumor efficacy of selinexor, a selective inhibitor of nuclear export, on breast cancer. We have shown that selinexor effectively inhibits cell proliferation and cell growth both in vitro and in vivo at biologically relevant drug concentrations*.* Our experiments suggest that there is enhanced sensitivity of TNBC cell lines to selinexor compared with ER+ cells. Treatment with selinexor as a single agent resulted in enhanced tumor growth inhibition in four of five TNBC PDX models in vivo. We also demonstrated that treatment with selinexor induced apoptosis. Concordantly, treatment with selinexor decreased levels of survivin, XIAP, and β-catenin. Furthermore, while survivin has been previously reported as a possible explanation for the mechanism of action of selinexor [3, 25], by testing a wide array of breast cancer cell lines in this study, we have shown that selinexor decreases XPO1 expression and survivin expression not only in selinexor-sensitive cell lines but also in selinexor-resistant lines. We have also shown that selinexor is effective independent of PTEN, PIK3CA, TP53, or Ras/Ras status. Although we did see greater sensitivity in TNBC cell lines compared to other subtypes, we have not tested the sensitivity of ER+ and HER+ cancers in vivo, and we have not determined the mechanism behind the differential sensitivity between subtypes in vitro. Further studies are needed to identify pharmacodynamic markers of response and mechanisms of intrinsic resistance. The inherent complexity of the mechanism behind XPO1 inhibition involves the ability of XPO1 to interact with several different tumor suppressors and cell cycle regulators, therefore potentially targeting multiple pathways. One of the most common examples is the role of XPO1 inhibition leading to the accumulation of TP53 in the nucleus, an exciting finding of a possible mechanism explaining its anti-proliferative effects in leukemia, lymphoma, prostate cancer, melanoma, and hepatocellular carcinoma [26–28]. In our study, however, most cell lines tested were *TP53* mutant yet differed in their selinexor sensitivity. Thus, XPO1 inhibition is effective independent of the TP53 status as we have shown here, consistent with what has been previously shown for sarcoma, non-small cell lung cancer, multiple myeloma, and mesothelioma [29–32].

Selinexor also had enhanced efficacy when combined with standard chemotherapy agents. Four different TNBC cell lines showed notable synergy in vitro, in particular with doxorubicin and paclitaxel (median CI values of 0.6 and 0.5, respectively), agents used commonly in the neoadjuvant or adjuvant treatment of TNBC, and to some extent with carboplatin and eribulin (median CI values of 0.8 and 0.7, respectively). At the same time, the combination of selinexor with paclitaxel enhanced apoptosis. Further, selinexor showed enhanced antitumor activity in vivo when combined with paclitaxel and eribulin, with T/C ratios <40% in three different TNBC PDX models.

## Conclusions

Altogether, these results provide preclinical evidence that selinexor is a promising agent in the treatment of TNBC, with enhanced antitumor activity in combination with chemotherapy. The safety and tolerability of selinexor combined with chemotherapy are being explored in ongoing clinical trials (NCT02419495), with planned expansions in breast cancer.

## Additional files

Additional file 1: PIK3CA, PTEN, TP53 and Ras/Raf status of cell lines in panel. A panel of breast cancer cell lines was tested with selinexor, and cell growth was measured after 72 hours of treatment using the SRB assay. IC_50_ was then calculated using isobologram curves. The mutation statuses for PIK3CA, PTEN, TP53, and Ras/Raf status are reported. (DOCX 23 kb)

Additional file 2: Effects of selinexor in breast cancer models. **A** Eight breast cancer cell lines were treated with vehicle or 5 μM selinexor. Cells were lysed and blotted with the indicated antibodies. **B** The bands were quantified and normalized to their respective β-actin controls. Data are shown as relative protein levels vs vehicle control of cell lines treated with selinexor. (DOCX 397 kb)

Additional file 3: Effects of selinexor on the cell cycle. SUM-159PT and MDA-MB-468 cells were treated with 1 nM paclitaxel alone (2 nM for MDA-MB-468), 400 nM selinexor alone, and the combination of both. After 72 hours, cells were stained with propidium iodide and analyzed via flow cytometry. (DOCX 514 kb)

Additional file 4: Actionable DNA alterations in PDXs. BCX 6, BCX 10, BCX 11, and BCX 22 were analyzed by targeted exome sequencing of 202 cancer-relevant targets and whole-exome sequencing. BCX 51 was analyzed using targeted exome sequencing of 265 genes that included XPO1. HDEL: copy number <1, HAMP: copy number >4. (DOCX 15 kb)

Additional file 5: Tolerance of selinexor in vivo. Body weight–time curve is shown. Data are presented as mean ± SEM. (DOCX 126 kb)

## Abbreviations

CI: Combination index
DMSO: Dimethyl sulfoxide
ER: Estrogen receptor
FACS: Fluorescence-activated cell sorting
IC_50_: Half-maximal inhibitory concentration
NES: Nuclear export sequence
PBS: Phosphate-buffered saline
PDX: Patient-derived xenografts
SRB: Sulforhodamine B
T/C ratio: Tumor growth inhibition ratio
TNBC: Triple-negative breast cancer
XPO: Exportin1 or CRM1
