# Supplementary material for: Selinexor (KPT-330) demonstrates anti-tumor efficacy in preclinical models of triple-negative breast cancer
Source: Breast Cancer Res. 2017 Aug 15;19:93. doi: 10.1186/s13058-017-0878-6 (PMC5557476; doi:10.1186/s13058-017-0878-6)
Supplement: Supplementary file 1 — PIK3CA, PTEN, TP53 and Ras/Raf status of cell lines in panel. A panel of breast cancer cell lines was tested with selinexor, and cell growth was measured after 72 hours of treatment using the SRB assay. IC50 was then calculated using isobologram curves. The mutation statuses for PIK3CA, PTEN, TP53, and Ras/Raf status are reported. (DOCX 23 kb) [file 13058_2017_878_MOESM1_ESM.docx]

**Additional file 1**

| **PIK3CA, PTEN, TP53 and Ras/Raf status of cell lines in panel** | | | | | |
| --- | --- | --- | --- | --- | --- |
| **Cell line** | **IC_50_ƞM** | **TP53** | **PTEN** | **PIK3CA** | **Ras/Raf** |
| MDA-MB-361 | 9765 | E166Stop |  | G1633A:E545K;  A1700G:K567R | |
| BT-474 | 1074 | E285K |  | G333C:K111N | |
| CAMA-1 | 53070 | A280T | G274C |  |  |
| HCC-1419 | 5216 | Del 6bp |  |  |  |
| MDA-MB-134vi | 1000 | E285K |  |  |  |
| MDA-MB-175 | 17001 | wt |  |  |  |
| BT-483 | 186337 | M246I |  | G1624A:E542K | |
| T-47D | 8823 | L194F |  | A3140G:H1047R | |
| MCF-7 | 39 | wt |  | G1624A:E542K;  G1633A:E545K | |
| SK-BR-3 | 774 | R175H |  |  | Q61R |
| HCC-1954 | 66 | Y163C |  | H1047R |  |
| HCC-1569 | 45 | E294Stop | K267fs |  |  |
| SUM-159PT | 11 | Ins 3a |  | H1047L | G12D |
| MDA-MB-231 | 12 | R280K |  |  |  |
| MDA-MB-436 | 16 | Ins 7bp | gross PTEN mutation | | |
| MDA-MB-468 | 24 | R273H | codon 70, 44 bp deletion,  frameshift | | |
| HCC-38 | 34 | R273L |  |  |  |
| BT-549 | 40 | R249S | codon 274, GTA AAT to  TAA AT, stop;  822delG:L295X | | |
| MDA-MB-453 | 47 | Del 30bp | G919A:E307K | A3140G:H1047R | |
| BT-20 | 50 | K132Q |  | C1616G:P539R;  A3140G:H1047R | |
| HCC-1143 | 62 | R248Q |  |  |  |
| HCC-1937 | 83 | R306Stop | gross PTEN mutation,  homozygous deletion | | |
| HCC-70 | 82 | R248Q | F90fs*9 |  |  |
| HCC-1806 | 29 | Ins 2bp |  |  |  |
| HCC-1395 | 148 | R175H | N212fs*1 |  |  |
| MDA-MB-157 | 550 | Del 26bp |  |  |  |

**Additional file 1.** **PIK3CA, PTEN, TP53 and Ras/Raf status of cell lines in panel.** Panel of breast cancer cell lines tested with selinexor, cell growth was measured after 72 hours of treatment using SRB assay and IC50 was then calculated using isobologram curves. Mutation status for PIK3CA, PTEN, TP53 and Ras/Raf status is reported [1-6].

1. Lacroix, M., R.A. Toillon, and G. Leclercq, *p53 and breast cancer, an update.* Endocr Relat Cancer, 2006. **13**(2): p. 293-325.

2. Meric-Bernstam, F., et al., *PIK3CA/PTEN mutations and Akt activation as markers of sensitivity to allosteric mTOR inhibitors.* Clin Cancer Res, 2012. **18**(6): p. 1777-89.

3. Sangai, T., et al., *Biomarkers of response to Akt inhibitor MK-2206 in breast cancer.* Clin Cancer Res, 2012. **18**(20): p. 5816-28.

4. Concin, N., et al., *Comparison of p53 mutational status with mRNA and protein expression in a panel of 24 human breast carcinoma cell lines.* Breast Cancer Res Treat, 2003. **79**(1): p. 37-46.

5. Lehmann, B.D., et al., *Identification of human triple-negative breast cancer subtypes and preclinical models for selection of targeted therapies.* J Clin Invest, 2011. **121**(7): p. 2750-67.

6. Saal, L.H., et al., *Recurrent gross mutations of the PTEN tumor suppressor gene in breast cancers with deficient DSB repair.* Nat Genet, 2008. **40**(1): p. 102-7.
