# Supplementary material for: Selinexor (KPT-330) demonstrates anti-tumor efficacy in preclinical models of triple-negative breast cancer
Source: Breast Cancer Res. 2017 Aug 15;19:93. doi: 10.1186/s13058-017-0878-6 (PMC5557476; doi:10.1186/s13058-017-0878-6)
Supplement: Supplementary file 2 — Effects of selinexor in breast cancer models. A Eight breast cancer cell lines were treated with vehicle or 5 μM selinexor. Cells were lysed and blotted with the indicated antibodies. B The bands were quantified and normalized to their respective β-actin controls. Data are shown as relative protein levels vs vehicle control of cell lines treated with selinexor. (DOCX 397 kb) [file 13058_2017_878_MOESM2_ESM.docx]

**Additional file 2**

**
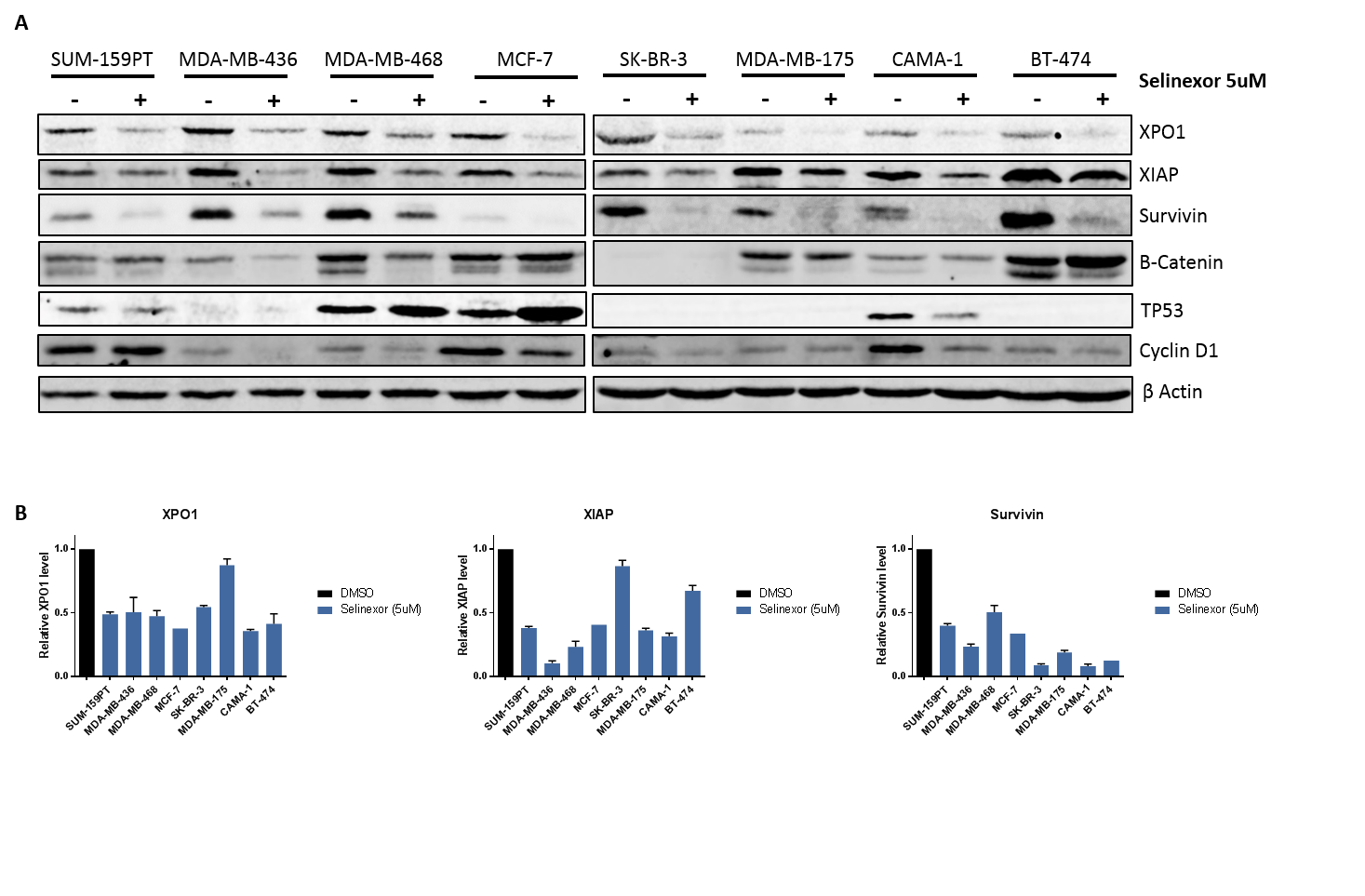
**

**Additional file 2.** **Effects of** **Selinexor in breast cancer models.** **A.** Eight breast cancer cell lines were treated with vehicle or 5µM Selinexor. Cells were lysed and blotted with the indicated antibodies. **B.** The bands were quantified and normalized to their respective β-actin control. Data is shown as relative protein levels vs vehicle control of cell line treated with selinexor.
