# Supplementary material for: Selinexor (KPT-330) demonstrates anti-tumor efficacy in preclinical models of triple-negative breast cancer
Source: Breast Cancer Res. 2017 Aug 15;19:93. doi: 10.1186/s13058-017-0878-6 (PMC5557476; doi:10.1186/s13058-017-0878-6)
Supplement: Supplementary file 3 — Effects of selinexor on the cell cycle. SUM-159PT and MDA-MB-468 cells were treated with 1 nM paclitaxel alone (2 nM for MDA-MB-468), 400 nM selinexor alone, and the combination of both. After 72 hours, cells were stained with propidium iodide and analyzed via flow cytometry. (DOCX 514 kb) [file 13058_2017_878_MOESM3_ESM.docx]

**Additional file 3**


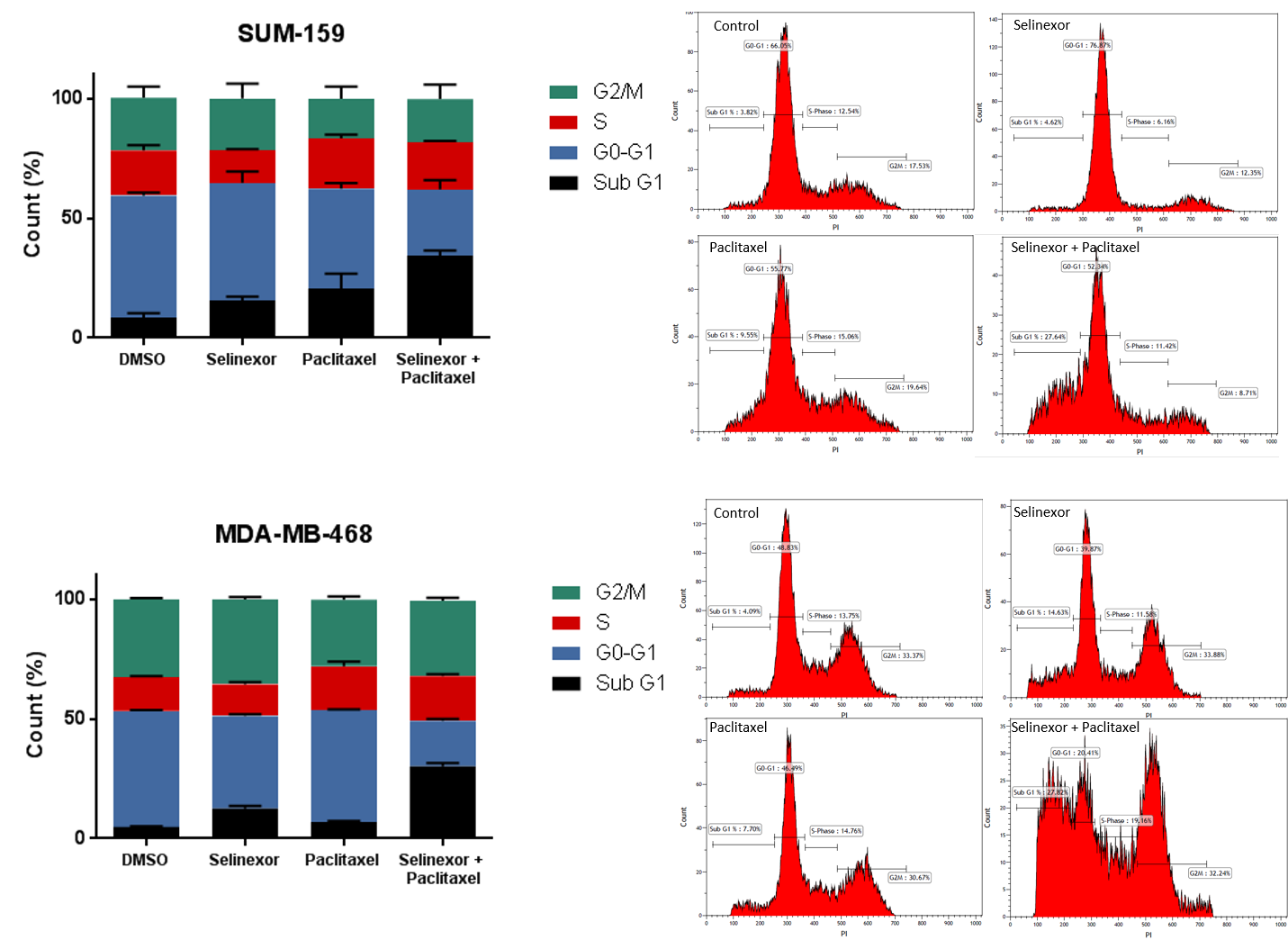


**Additional file 3.** **Effects of Selinexor on cell cycle.** SUM-159PT and MDA-MB-468 cells were treated with 1nM paclitaxel alone (2nM for MDA-MB-468), 400nM selinexor alone, and combination of both. After 72 hours, cells stained with PI and analyzed via flow cytometry.
