# Supplementary material for: Selinexor (KPT-330) demonstrates anti-tumor efficacy in preclinical models of triple-negative breast cancer
Source: Breast Cancer Res. 2017 Aug 15;19:93. doi: 10.1186/s13058-017-0878-6 (PMC5557476; doi:10.1186/s13058-017-0878-6)
Supplement: Supplementary file 4 — Actionable DNA alterations in PDXs. BCX 6, BCX 10, BCX 11, and BCX 22 were analyzed by targeted exome sequencing of 202 cancer-relevant targets and whole-exome sequencing. BCX 51 was analyzed using targeted exome sequencing of 265 genes that included XPO1. HDEL: copy number <1, HAMP: copy number >4. (DOCX 15 kb) [file 13058_2017_878_MOESM4_ESM.docx]

**Additional file 4**

| **Table S2. Selected DNA alterations in the PDX models** | |
| --- | --- |
| **PDX** | **DNA Alterations** |
| BCX 06 | PI3KCA H1047R, FGFR4 HAMP, NOTCH2 HAMP, BRCA1/2 WT, TP53 WT, XPO1 WT |
| BCX 10 | PI3KCA H1047R, PTEN HDEL, BRCA1/2 WT, TP53 Q331, XPO1 WT |
| BCX 11 | FGFR1 HAMP, BRCA1/2 WT, TP53 R273H, XPO1 WT |
| BCX 22 | ATM HDEL, BRCA1/2 WT, TP53 E221, XPO1 WT |
| BCX 51 | STK11 HDEL, BRCA1/2 WT, TP53 C124, XPO1 WT. |

**Additional file 4. Actionable DNA alterations in PDXs**. BCX6, BCX10, BCX11 and BCX22 were analyzed by targeted exome sequencing of 202 cancer-relevant targets and whole exome sequencing. BCX 51 was analyzed using targeted exome sequencing of 265 genes that included XPO1. HDEL: Copy number <1, HAMP: Copy number >4.
