# Supplementary material for: Selinexor (KPT-330) demonstrates anti-tumor efficacy in preclinical models of triple-negative breast cancer
Source: Breast Cancer Res. 2017 Aug 15;19:93. doi: 10.1186/s13058-017-0878-6 (PMC5557476; doi:10.1186/s13058-017-0878-6)
Supplement: Supplementary file 5 — Tolerance of selinexor in vivo. Body weight–time curve is shown. Data are presented as mean ± SEM. (DOCX 126 kb) [file 13058_2017_878_MOESM5_ESM.docx]

**Additional file 5**


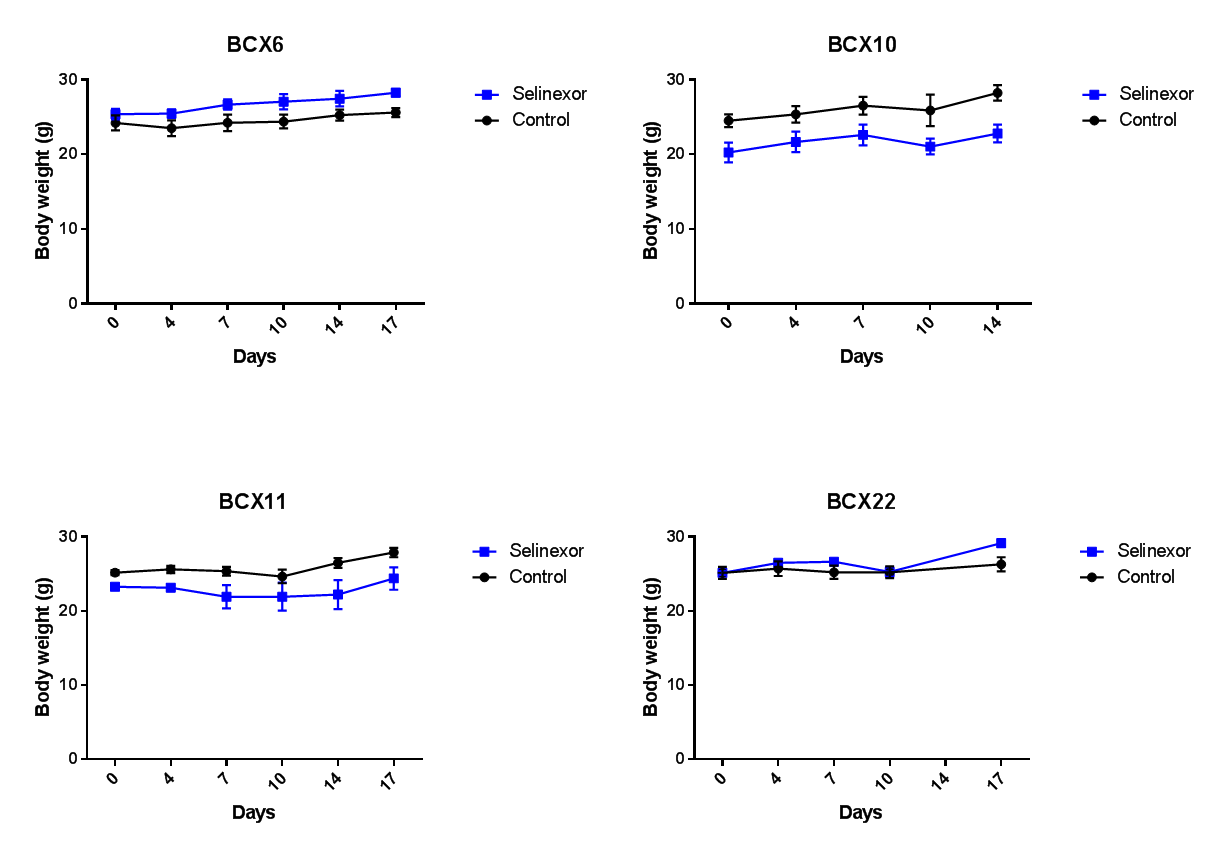


**Additional file 5.** **Selinexor is well tolerated in *in vivo* models.** Body weight-time curve is shown. Data is presented as mean ±SEM.
